# Analysis of complex chromosomal rearrangements using a combination of current molecular cytogenetic techniques

**DOI:** 10.1186/s13039-022-00597-y

**Published:** 2022-05-19

**Authors:** Ping He, Xiaoni Wei, Yuchan Xu, Jun Huang, Ning Tang, Tizhen Yan, Chuanchun Yang, Kangmo Lu

**Affiliations:** 1 Department of Medical Genetics, Liuzhou Maternal and Child Health Hospital, Liuzhou, Guangxi China; 2 CheerLand Biological Technology Co., Ltd., Shenzhen, China; 3 Prenatal Diagnosis Center, Affiliated Foshan Maternity & Child Healthcare Hospital, Southern Medical University (Foshan Maternity & Child Healthcare Hospital), Foshan, Guangdong China

**Keywords:** Complex chromosome rearrangements (CCR), Karyotype analysis, Fluorescence in situ hybridization (FISH), High-throughput whole-genome sequencing (WGS)

## Abstract

**Background:**

Using combined fluorescence in situ hybridization (FISH) and high-throughput whole-genome sequencing (WGS) molecular cytogenetic technology, we aim to analyze the junction breakpoints of complex chromosome rearrangements (CCR) that were difficult to identify by conventional karyotyping analysis and further characterize the genetic causes of recurrent spontaneous abortion.

**Results:**

By leveraging a combination of current molecular techniques, including chromosome karyotype analysis, FISH, and WGS, we comprehensively characterized the extremely complex chromosomal abnormalities in this patient with recurrent spontaneous abortions. Here, we demonstrated that combining these current established molecular techniques is an effective and efficient workflow to identify the structural abnormalities of complex chromosomes and locate the rearrangement of DNA fragments.

**Conclusions:**

In conclusion, leveraging results from multiple molecular and cytogenetic techniques can provide the most comprehensive genetic analysis for genetic etiology research, diagnosis, and genetic counseling for patients with recurrent spontaneous abortion and embryonic abortion.

## Background

Complex chromosomal rearrangement (CCR) is a special type of abnormal chromosome structure that involves two or more chromosomes and three or more breakpoints [1–6]. The primary type of CCR is chromosomal translocation, which may also be accompanied by chromosome inversion and fragment insertion. Simple reciprocal translocation involving three chromosomes is the most common. The incidence of somatic peripheral blood cell CCR in the general population was extremely low. There were currently 255 cases reported in the world [7], of which 124 cases were carriers of chromosome translocation [8]. Most CCRs are de novo, and very few come from parents [9].

Approximately 70% of CCR carriers do not have any significant clinical symptoms [10], but recurrent miscarriage, embryonic abortion, multiple congenital malformations, and other reproductive issues have been reported. However, a small portion of CCR carriers can exhibit specific abnormalities, such as multiple malformations, intellectual disabilities, and growth retardation.

Conventional chromosome banding and chromosome karyotype analysis techniques are considered the gold standards for detecting chromosome structural abnormalities. These methods are less technically challenging and cost-effective ways to identify large fragments of chromosomal aberrations. However, for small fragments or abnormal chromosomal fragments with unclear bands, even the high-resolution chromosome analysis technology is insufficient to determine the source of minor abnormalities, let alone the accompanied microdeletion or microduplication. Fluorescence in situ hybridization (FISH) technology, especially the use of subtelomere probe technology, could detect some complex chromosomal aberrations while also providing an overview of the complete chromosome karyotype [11]. Conventional cytogenetic analysis technology combined with fluorescence in situ hybridization technology has been widely used in the chromosomal analysis. However, traditional cytogenetic analysis is limited by its resolution and often impossible to accurately localize chromosomal break sites and identify microdeletions or microduplications. These limitations have prompted combining traditional cytogenetics techniques with more sophisticated detection techniques. WGS based on NGS technology has a comprehensive coverage and could obtain complete and diverse genome information. It has its unique advantages in detecting structural chromosomal variation. It can accurately detect chromosomal break sites and chromosomal abnormalities, when combined with traditional cytogenetic analysis.

This article combined the conventional chromosome karyotype analysis, fluorescence in situ hybridization, and high-throughput whole-genome sequencing technologies to analyze complex chromosome structural abnormalities involving multiple chromosome rearrangements and abnormal chromosomal fragments.

## Results

### Chromosome G banding karyotype analysis

**Fig. 1:**
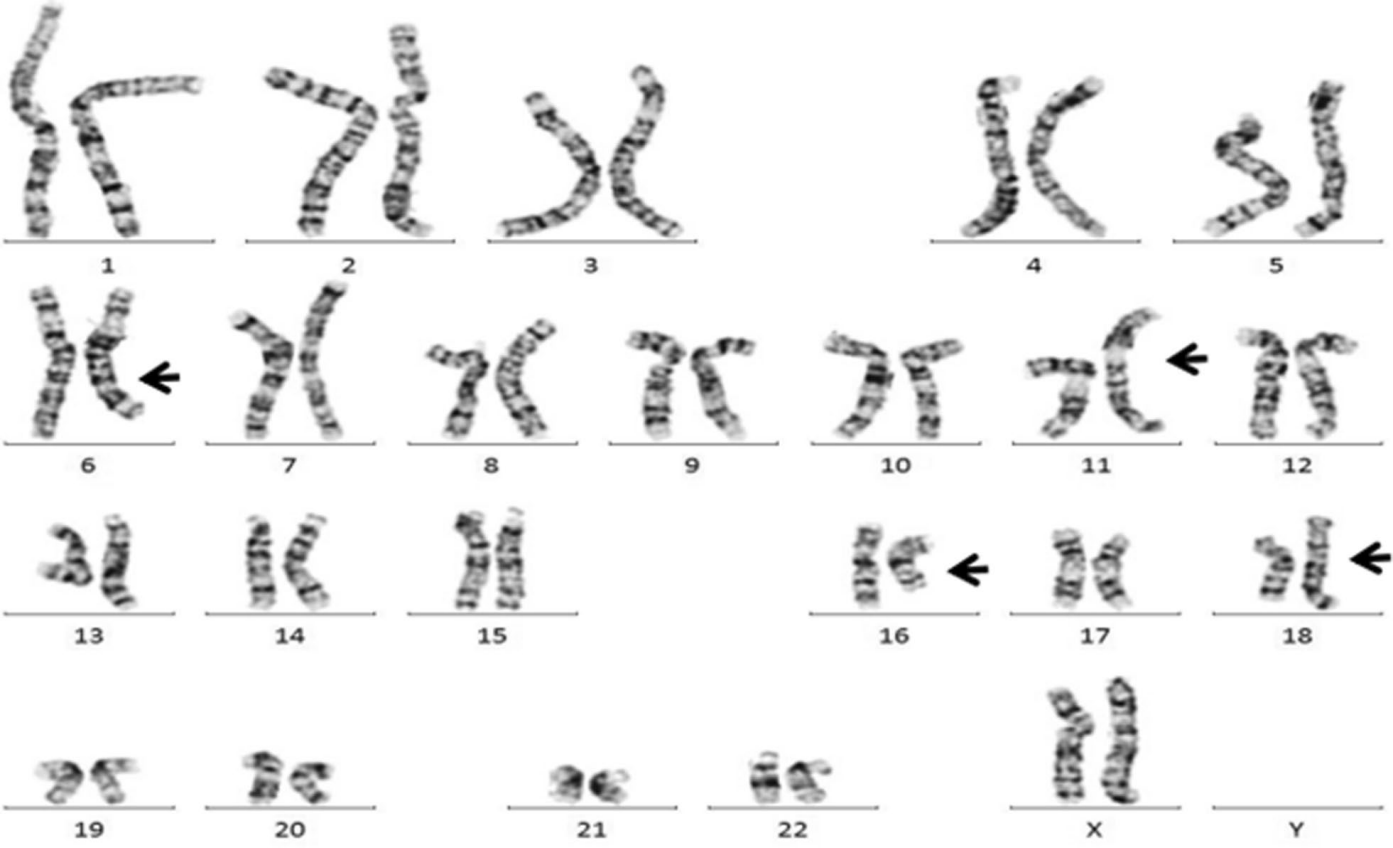
Chromosome karyotyping of the proband. The proband's chromosome number was normal,but multiple autosomal structural variations were detected: der(6)t(6;11)(q?;p?),inv(6)(q?q?), der(11)t(6;11)(q?;p?)inv(11)(p?q?), der(16)t(16;18)(q?;p?), der(18)t(16;18)(q?;p?)inv(18)(p?q?),inv(18)(q?q?), and insertion of chromosome fragments of unknown origin

Karyotype analysis showed that the patient’s chromosome number was normal, but multiple autosomal structural variations were detected, including translocations between the long arm of chromosome 6 and the short arm of chromosome 11, insertion of chromosome fragments of unknown origin on the long arm of chromosome 6, inversion between the arms of chromosome 11, and translocations between the long arm of chromosome 16 and the short arm of chromosome18 arm, pericentric and paracentric inversions of chromosome 18, and insertion of chromosome fragments of unknown origin (Fig. 1). The proband’s mother’s karyotype was 46, XX, and the father’s karyotype was 46, XY, which were normal karyotypes with no apparent abnormalities. Therefore, the chromosome structure variation of the proband was de novo.

## FISH analysis

**Fig. 2:**
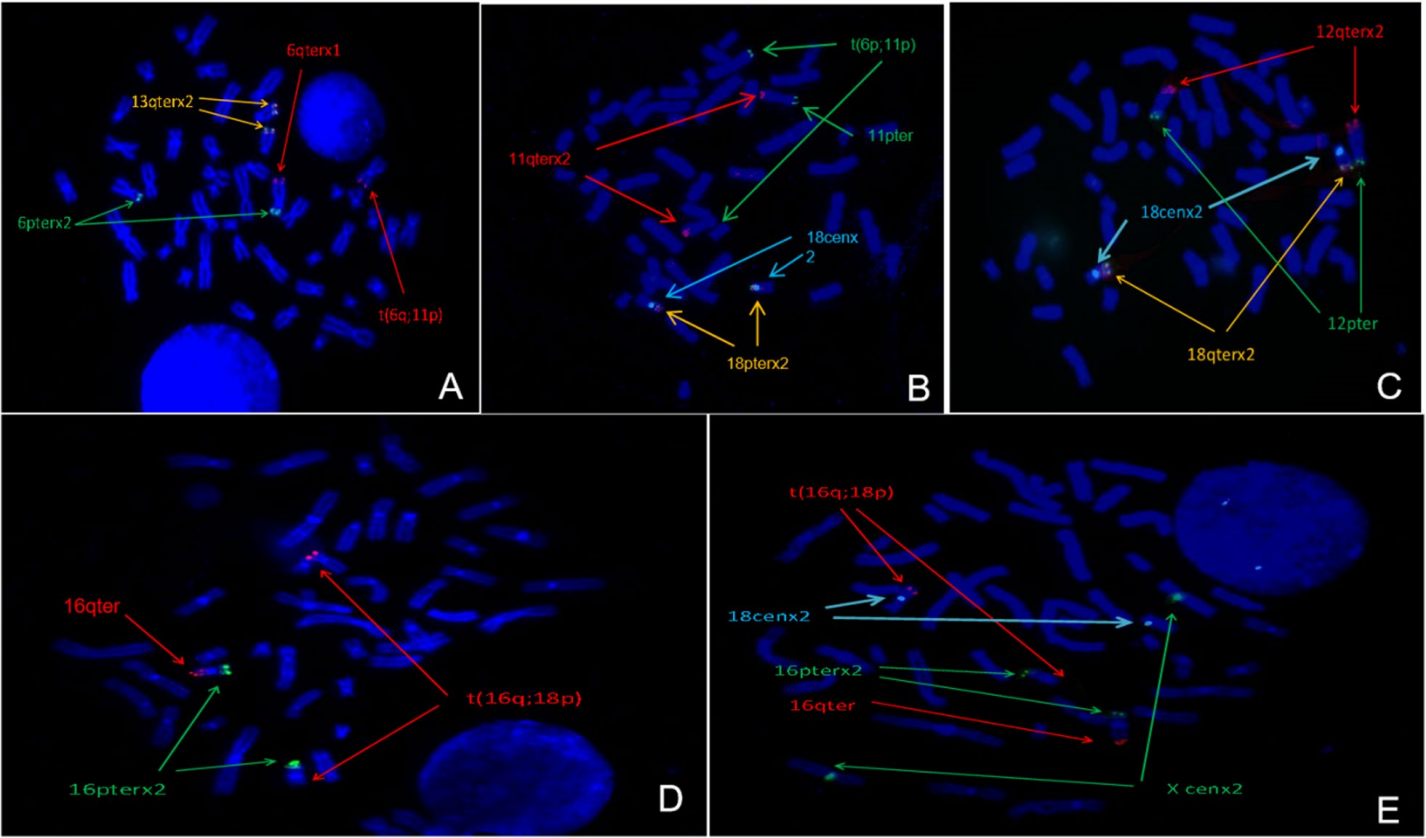
FISH analysis of the proband. **A** mixed probes of p + q sub-telomeres of chromosome 6 (as shown as green and red)and q sub-telomeres of chromosome 13(as shown as orange) showed that the end of the long arm of chromosome 6 was shifted to chromosome 11. **B** mixed probes of p+q sub-telomeres of chromosome 11 (as shown as green and red), centromeric and p sub-telomere of chromosome 18(as shown as cyan and orange) showed that one of the short arms of chromosome 11 was shifted to chromosome 6, and **A**+**B** proved that the long arm of chromosome 6 and the short arm of chromosome 11 were interchanged, as “ish t(6;11)(q27−,p15+ ;p15−,q27+)”, **C** mixed probes of chromosome 12 p+q terminal (as shown as green and red) and 18p terminal and sub-telomere (as shown as orange and cyan) showed that no abnormalities at the end of the long arm of chromosome 18, **D**+**E** proved that one of the long arm of chromosome 16 shifted to the end of the short arm of chromosome 18, as “ish t(16;18)(q24−,p11. 3+ ; p11.3-,q24+)”

To determine the sites of reciprocal translocation, cells in metaphase from the proband's peripheral blood lymphocytes by FISH were analyzed. FISH results were as shown in Fig. 2. Those of mixed probe #1 showed that the end of the long arm of chromosome 6 was shifted to chromosome 11 (Fig. 2A); mixed probe #2 showed that one of the short arms of chromosome 11 was shifted to chromosome 6 (Fig. 2B); the results of Fig. 2A+B proved that the long arm of chromosome 6 and the short arm of chromosome 11 were interchanged as "ish t(6;11)(q27-,p15+ ;p15-,q27+)"; mixed probe #3 showed no abnormalities at the end of the long arm of chromosome 18 (Fig. 2C); FISH of mixed probes #4 and #5 both showed that one of the long arm of chromosome 16 shifted to the end of the short arm of chromosome 18 (Fig. 2 D&E), resulting in ish t(16;18)(q24−,p11. 3+ ; p11.3−,q24+).

### Whole-genome high-throughput sequencing analysis

**Fig. 3:**
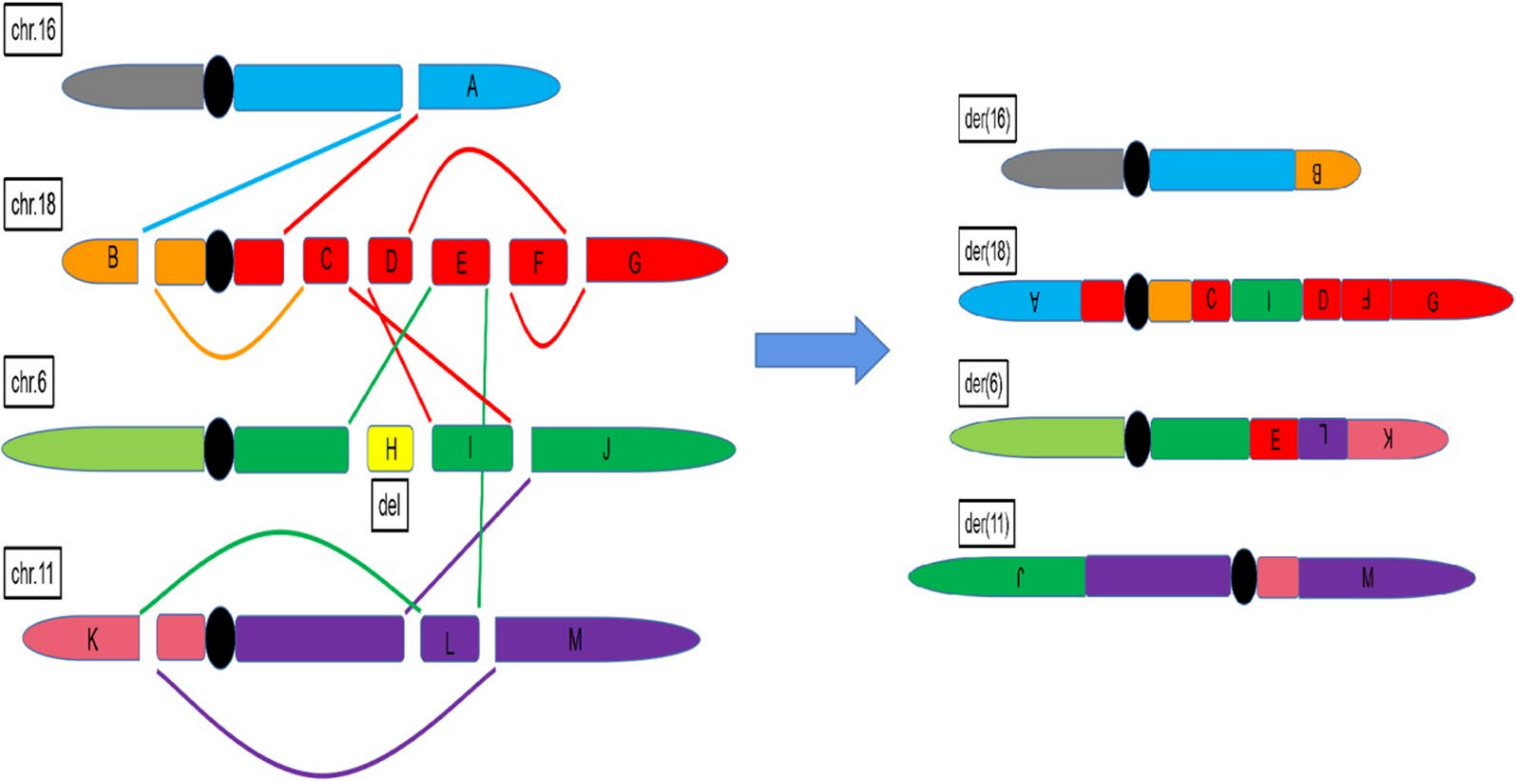
The schematic of chromosome rearrangement.Chromosomes 6p and 6q are indicated as pale green and green, respectively, 2.2 Mb deletion on chromosome 6 is indicated as yellow, 11p and 11q are indicated as pink and purple, 16p and 16q are indicated as gray and cyan, and 18p and 18q are indicated as orange and red. It showed that chromosome 6q and 11p were interchanged, 16q shifted to the end of 18p, 11p and 11q were interchanged, 18p and 18q were interchanged, 18p and 18p were interchanged, 6q and 18q were inserted into each other and 2.2 Mb deletion on chromosome 6

The complex structural rearrangement of the proband's chromosome could not be completely and accurately described with only conventional chromosome karyotype G banding combined with FISH analysis. Therefore, it was necessary to use WGS to further analyze the position of the breakpoints of rearranged chromosomes on the derived chromosomes. The results of high-throughout sequencing and bioinformatics analysis of peripheral blood genomic DNA revealed 3 breakpoints on chromosome 6, including 6q22.31 (122,832,871), 6q22.31 (125,055,682), 6q23.3 (138,438,243); 2.2 Mb deletion including 8 genes of *ATP5LP2、CLVS2、FABP7、NKAIN2、PKIB、RN7SL564P、SMPDL3A、TRDN* on 6q22.31; 3 breakpoints on chromosome 11 including 11p14.3(25,427,432), 11q14.1 (82,431,656), 11q14.1 (84,346,157); 1 breakpoint on chromosome 16 including16q22.1(68,295,707); 6 breakpoints on chromosome 18 including 18p11.31(4,265,706), 18q12.2(33,329,662), 18q21.1(46,251,913), 18q21.2 (48,694,348), 18q21.31(54,118,504), 18q22.1(63,838,338), as shown in Table 1. There were complex rearrangements between chromosomes 6, 11, 16, and 18 (as shown in Fig. 3), involving breaks of 4 genes, namely*PLA2G15, DLG2, DLGAP1-AS5*, and *CTIF*.Then, align the high-quality reads to the human reference genome (hgl9, GRCh37.1), complex rearrangements are described as seq[hgl9]del(6)(q22.31q22.31)ins(6;18;11)(q22.31;q21.2q21.1;q14.1q14.1)t(6;11)(q22.31;p14.3)[g.pter_122832871:g.48694348_54118504:g.84346157:82431656inv:g.25427432_pter],seq[hgl9]inv(11)(p14.3q14.1)[g.qter_138438243:g.82431656_25427432inv:84346157_qter],seq[hgl9]t(16;18)(q22.1;p11.3)[g.pter_68295707:g.4265706_pter],seq[hgl9]inv(18)(p11.31q12.2)ins(18;6)(q21.1;q23.3q22.31)inv(18)(q21.31q22.1)[g.qter_68295707:g.33329662_4265706inv:33329662_46251913:g.138438243_125055682inv:g.46251913_48694348:63838338_54118504inv:63838338_qter].

**Table 1:** The results of high-throughout sequencing and bioinformatics analysis of peripheral blood genomic DNA. It showed that 13 breakpoints on chromosome 6, 11,16 and 18. There were 3 breakpoints on chromosome 6, including 6q22.31 (122,832,871), 6q22.31 (125,055,682), 6q23.3 (138,438,243) and 2.2 Mb deletion; 3 breakpoints on chromosome 11, including 11p14.3 (25,427,432), 11q14.1 (82,431,656), 11q14.1 (84,346,157); 1 breakpoint on chromosome 16 including16q22.1(68,295,707); 6 breakpoints on chromosome 18 including 18p11.31 (4,265,706), 18q12.2 (33,329,662), 18q21.1 (46,251,913), 18q21.2 (48,694,348), 18q21.31 (54,118,504), 18q22.1 (63,838,338). And 2.2 Mb deletion on chromosome 6 included 8 genes of *ATP5LP2*, *CLVS2*, *FABP7*, *NKAIN2*, *PKIB*, *RN7SL564P*, *SMPDL3A*, *TRDN* on 6q22.31; there were complex rearrangements between chromosomes 6, 11, 16, and 18, involved breaks of 4 genes, namely*PLA2G15, DLG2, DLGAP1-AS5*, and *CTIF*

Breakpoint	Gene
6q22.31(122,832,871)	–
6q22.31(125,055,682)	–
6q23.3(138,438,243)	–
11p14.3(25,427,432)	–
11q14.1(82,431,656)	–
11q14.1(84,346,157)	*DLG2*
16q22.1(68,295,707)	*PLA2G15*
18p11.31(4,265,706)	*DLGAP1-AS5*
18q12.2(33,329,662)	–
18q21.1(46,251,913)	*CTIF*
18q21.2(48,694,348)	–
18q21.31(54,118,504)	–
18q22.1(63,838,338)	–
6q22.31(122,832,871–125,055,682)	*ATP5LP2,CLVS2,FABP7,NKAIN2,PKIB,RN7SL564P,SMPDL3A,TRDN*

### Overview of the complete karyotype of the proband

In summary, based on conventional cytogenetics and molecular genetics, and in accordance with the ISCN2020 chromosome naming principles, the patient's complete chromosome karyotype was described as follows:

46,XX,del(6)(q22.31q22.31)ins(6;18;11)(q22.31;q21.2q21.1;q14.1q14.1)t(6;11)(q22.31;p14.3),inv(11)(p14.3q14.1),t(16;18)(q22.1;p11.3),inv(18)(p11.31q12.2)ins(18;6)(q21.1;q23.3q22.31)inv(18)(q21.31q22.1) dn. After inquiring the existing databases worldwide, no report was found about that was completely consistent with the karyotype and sequencing results of the proband studied in this study. It was the first report of non-tumor somatic cell with such a complex chromosomal structure abnormality in a patient with only recurrent spontaneous abortions, per authors’ knowledge.

## Discussion

Complex chromosomal rearrangements are very rare in the general population. Researchers had proposed many classification methods for CCRs according to the significance and impact of CCRs [12]. In general, tripartite rearrangement CCRs accounted for the majority, followed by double reciprocal translocation CCRs, and both generally involved only chromosomal translocations. There were usually multiple breakpoints in special CCRs, and CCRs could produce multiple derivative chromosomes. They often involved chromosomal translocations and accompanied by structural abnormalities such as chromosomal inversions, insertions, and deletions. The case reported in this article was the only case with as many as 13 breakpoints, including special CCRs with reciprocal translocations, inversions, insertions and deletions reported to date in the literature. These involved structural abnormalities on the four chromosomes 6, 11, 16, and 18.

The mechanism of CCRs is very complex, and it has not yet been fully characterized. Studies had reported that the rearrangement of chromosomal structure was essentially due to double-strand breaks (DSBs) and double-strand break repair [13]. A variety of endogenous and exogenous factors could damage chromosomes and caused DNA double-strand breaks including but not limited to ionizing radiation, radiotherapy, chemotherapy, free radical damage, and viral infection [14]. The failure to repair double-strand breaks in time may be the direct cause of chromosomal structural rearrangement. The occurrence of CCRs is random, involving multiple fracture points and multiple regions. A single fractural rearrangement cannot produce CCRs. Pellestor et al. [7] has proposed that the formation of CCRs was likely not caused by simple gene point mutations, but by the structural characteristics of the genome. In this case of special CCRs, there was no clear radioactivity, drug, chemical exposure history, nor history of bacterial virus infection and other exogenous in-utero toxic exposure. Other members of the family had no abnormal chromosomal structure, and thus the cause of proband’s complex CCRs was unclear.According to the current data and internal and external research reports, the molecular mechanism of CCR formation can not be clarified, and greater data collection and further research may be needed.

In this study, relying on traditional cytogenetic analysis techniques, we identified 6q abnormality, chromosome 11 inversion, reciprocal displacement of chromosomes 16 and 18, and 18q abnormality. However, due to the low resolution of this technology and the inability to distinguish similar bands, it cannot accurately characterize the complete karyotype abnormalities of CCR carriers. In order to further clarify the genetic aberrations, we performed fluorescence in situ hybridization analysis of subtelomere probes related to chromosomal abnormalities based on conventional karyotype analysis. The results showed t(6q;11p), t(16q;18p), but the specific form of 18q abnormality was still not clear. According to the results of high-throughput sequencing of the whole genome, it was found that the chromosome structure variation was very complicated, not only t(6q;11p)inv(11), t(16q;18p), but also some unresolved or unrecognized aberrations in G banding or FISH detection and analysis, such as partial deletion of 6q, and partial insertion of chromosome 18 and 11 into the long arm of chromosome 6. The same chromosome 18 has both inter-arm inversion and intra-arm inversion, specifically involving chromosome 6, 11, 16 and 18. There were 13 breakpoints, and the types of distortion included reciprocal translocation, intra-arm inversion, inter-arm inversion, insertion, deletion and so on. The complex rearrangements between chromosomes 6, 11, 16, and 18 involved the breaks of *PLA2G15, DLG2, DLGAP1-AS5*, and *CTIF* genes. Genetic diseases associated with these genes had not been included in OMIM. There was about 2.2 M deletion in the q22.31 region of chromosome 6, involving 8 genes (*ATP5LP2, CLVS2, FABP7, NKAIN2, PKIB, RN7SL564P, SMPDL3A, and TRDN)*. The *TRDN* gene was related to autosomal recessive catecholamine-sensitive ventricular tachycardia type 5 with or without myasthenia (OMIM:615,441), but it was generally caused by homozygous or compound heterozygous mutations. No spontaneous abortion has been reported for the involved gene mutations. The genes involved by chromosomal aberrations in this carrier were not directly associated with its spontaneous abortion.Although about 70% of CCR carriers did not have any clinically evident phenotype in the population test, they often caused many problems give rise on infertility [15], such as azoospermia, oligospermia, infertility, recurrent miscarriage, embryonic abortion, stillbirth, birth of congenital multiple deformities. Among them, the incidence of spontaneous abortion and embryo abortion was the highest. Madan et al. [12], Gorski et al. [16] estimated that the incidence of spontaneous abortion and embryonic abortion in CCR carriers was about 50%. Studies by Yaping Liao [8] and Peng Dai et al. [17] showed that the incidence of spontaneous abortion and embryo abortion were 77.6% and 81.6%, respectively, which was significantly higher in China than the rest of the world. Although the pregnancy outcome may vary greatly depending on ethnicities and geographic locations, studies worldwide had shown that the risk of abnormal pregnancy in CCRs carriers was extremely high. Special complex carriers of CCR had the highest risk of spontaneous abortion and embryo abortion [17]. In the patients involved in this study, 4 natural conceptions were aborted in twelve weeks. The main reason was that the patient’s chromosomes involved complex chromosomal rearrangements with multiple chromosomes, multiple sites, and multiple types of aberrations. During the meiosis process of egg formation in this patient, the probability of forming normal or balanced gametes was extremely low after the 4 chromosomes involved in structural rearrangement undergo pairing between homologous chromosomes and after meiosis. Most of them would form partial monomers or partial trisomy or even more complex unbalanced gametes, leading to early miscarriage, embryonic abortion, and stillbirth after fertilization. The probability that the patient could naturally give birth to normal offspring was extremely low. Although the rapid development of third-generation IVF assisted reproductive technology in recent years could help CCR carriers with low fertility to give birth successfully [18–23], the probability of obtaining normal or balanced embryos was very low and expensive. It may cause great harm to the patient and his family both physically and mentally. At this stage, if conditions permit, egg donation or adoption may be more realistic.

## Conclusions

In summary, we demonstrated the application of conventional karyotyping combined with fluorescence in situ hybridization and whole-genome high-throughput sequencing molecular cytogenetics methods can effectively identify occult causes of recurrent miscarriage. The reason in this case was mainly the special CCRs with 13 breakpoints involving 4 chromosomes (6, 11, 16 and 18). Subtelomere metaphase cells fluorescence in situ hybridization and whole-genome high-throughput sequencing could accurately locate chromosomal breakpoints [24, 25], and detect chromosomal aberrations and gene copy number variations that could not be detected by traditional cytogenetic analysis techniques. The application of conventional cytogenetics technology combined with whole-genome high-throughput sequencing in the clinic, has greatly facilitated the research on the genetic causes of complex chromosomal rearrangements such as the history of recurrent miscarriage, the history of embryonic abortion, and the history of childbirth with multiple congenital malformations. This will ultimately help greatly improve diagnostic efficiency and obtain better eugenic genetic counseling.

## Methods

### Case presentation

**Fig. 4:**
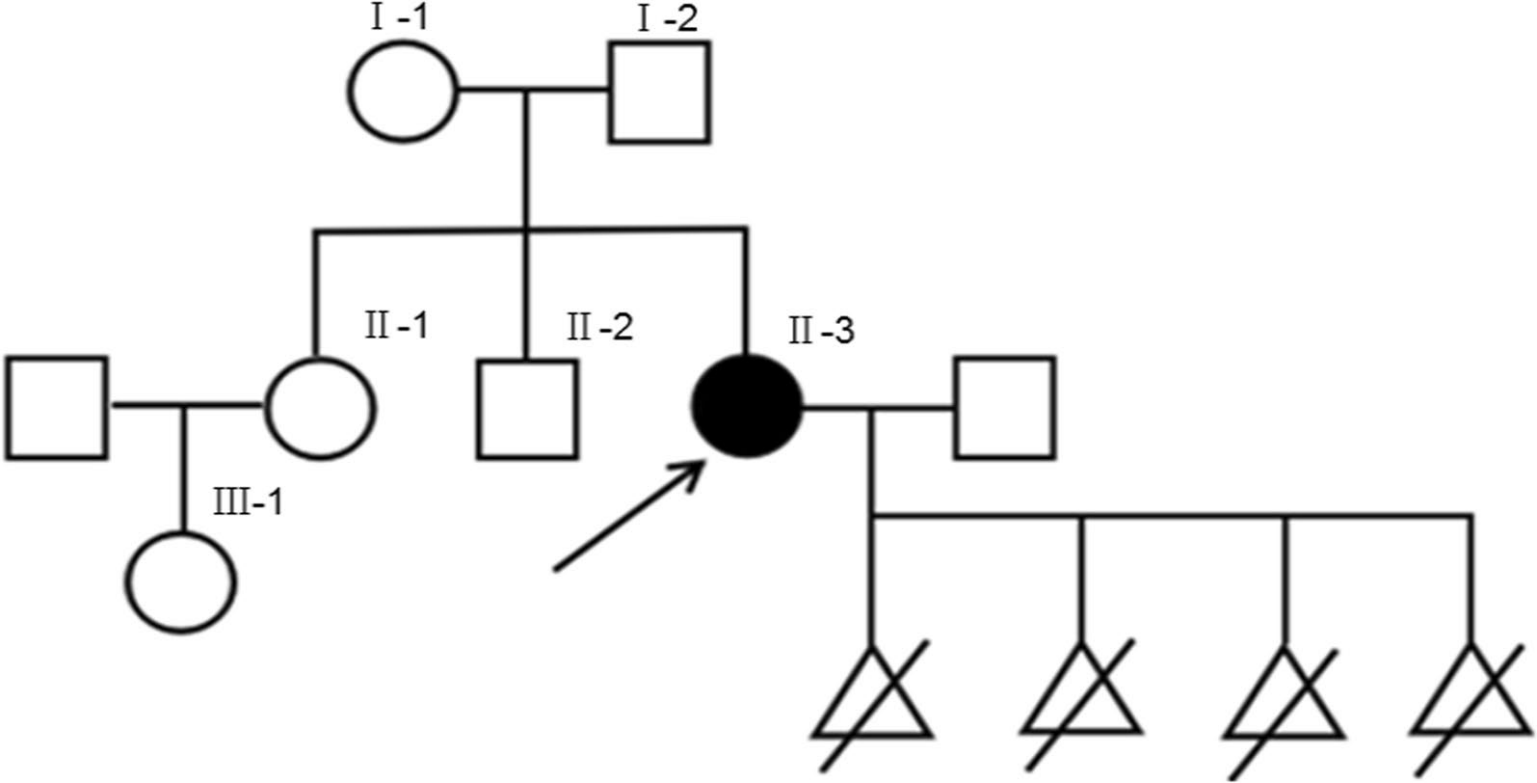
Pedigree of proband and her family. I represents the proband’s parents; II represents the proband and her sister and brother; II-3 is the proband; III-1 is the proband’s niece

The patient (proband) is a 30-year-old G4P0 woman, who presented to the hospital for menstrual irregularities and recurrent spontaneous abortion. The patient denies any radiation and toxin exposure during pregnancy. Clinical examinations revealed normal growth and development, no intellectual disabilities, normal hearing and vision, no facial features and limb deformities. Review of systems showed no abnormalities. Routine semen examination of the husband was normal, and no consanguinity has been reported in the family. The known pedigree of proband is shown in Fig. 4. Basic chemistry, immunological panel, and other relevant laboratory testing revealed no abnormalities.

### Karyotype analysis

Peripheral blood chromosomal analysis was performed on the proband and her parents. Five mL of peripheral venous blood was collected in a vacuum tube with sodium heparin and then gently mixed, followed by conventional peripheral blood culture, metaphase cell preparation, and conventional chromosome G banding. The G-banded slides were fully automated metaphase cell chromosomes; we screened the metaphase cells at the level of 450–500 bands. The cells in metaphase with clear bands were selected for chromosome karyotype analysis using Zeiss’s automatic chromosomal analysis system.

### Fluorescence in situ hybridization

To determine the reciprocal translocation positions on the derived chromosomes, the slides of peripheral blood metaphase cells without G banding were prepared and respectively hybridized with the following 5 sets of mixed probes from Vysis for fluorescence in situ hybridization analysis. Mixed probes were (1) p+q sub-telomeres of chromosome 6 and q sub-telomere of chromosome 13; (2) p+q sub-telomeres of chromosome 11, Centromeric, and p sub-telomere of chromosome 18; (3) p+q sub-telomeres of chromosome 12 and p sub-telomere of chromosome 18; (4) p+q sub-telomeres of chromosome 16; (5) p+q sub-telomeres of chromosome 16 and centromeres of chromosome 18, X and Y.

### High-throughout whole-genome sequencing

#### Sample processing

The genomic DNA was extracted from 5.0 ml of peripheral blood from the proband in EDTA using QIAamp DNA mini kit according to the manufacturer’s protocol (Qiagen, Hilden, Germany). The quality and concentration of the extracted DNA were evaluated using Invitrogen's Qubit 3.0 Fluorometer (Invitrogen, Carlsbad, CA, USA) and NanoDrop-One (Thermo Scientific, Wilmington, DE, USA) per the manufacture’s protocol. DNA samples were then stored at  − 20 °C.

#### Library construction and sequencing

To obtain small fragments of 200–500 bp DNA, 1.0 ug of genomic DNA was sonicated in Covaris M220 focused ultrasound instrument according to the manufacturer’s operating procedures. DNA fragments between 100 and 300 bp were selected with AMPure XP beads (Agencourt, CA, USA). DNA ends were ligated with a base addition and adaptors provided by the kit per the manufacturer’s protocol. Then, eight cycles of PCR pre-amplification were performed. DNA fragments with a length of about 500 bp were extracted from a 2% agarose gel after electrophoresis and finally obtained in a single-stranded circular DNA library.

Agilent Bioanalyzer DNA 2100 Kit (Agilent, CA, USA) and Qubit 3.0 (Invitrogen, Carlsbad, CA, USA) were used to ensure the quality of the constructed library. IlluminaNoaSeq platform was used for massively parallel sequencing for libraries that passed QC.

### Sequencing data analysis

The sequencing data was generated using the IlluminaNoaSeq platform. SAOPnuke (version 1.5.0) was used to filter the data, including the tags, adaptor sequences, and low-quality (containing more than five unidentified bases) reads per the manufacturer’s protocol. The SOAP2 software was used to align the high-quality reads to the human reference genome (hgl9, GRCh37.1) [24, 25], and then the unique aligned reads (Unique Reads) were isolated for subsequent analysis.

Finally, the breakpoints were validated by junction-spanning PCR.

## Data Availability

The datasets used and/or analyzed during the current study are available from the corresponding author on reasonable request.

## Abbreviations

CCR: Complex chromosome rearrangements
FISH: Fluorescence in situ hybridization
WGS: High-throughput whole genome sequencing
NGS: Next generation sequencing
